# Supplementary material for: Assessing the relationship between digital technology use and physical health, fitness, and exercise levels among Chinese youth: The moderating effect of parental monitoring
Source: PLoS One. 2025 Jun 4;20(6):e0324705. doi: 10.1371/journal.pone.0324705 (PMC12136296; doi:10.1371/journal.pone.0324705)
Supplement: Attachment — Submitted filename: Assesing.....pdf [file pone.0324705.s004.pdf]

Strengths of the Manuscript:

1. Relevance and Importance
    - The research tackles a pressing issue—digital technology use and its relationship with physical health among Chinese youth—an area of growing global concern. The focus on Chinese youth adds a unique cultural perspective to the discourse.
    - The inclusion of parental monitoring as a moderating factor and youth attitudes as mediators enriches the scope of the study, offering practical and theoretical insights.

2. Methodological Rigor
    - The study uses robust statistical techniques (e.g., regression, moderation, mediation analyses) and validated scales (e.g., PAQ-A, ATPA) to ensure reliability and validity.
    - The sample size of 827 respondents across diverse regions in China is commendable, ensuring generalizability.

3. Findings and Implications
    - The moderation and mediation analyses add depth, providing actionable insights for parents, educators, and policymakers.
    - The results emphasize the roles of parental involvement and positive attitudes towards physical activity, which have direct implications for designing interventions.

4. Clarity and Organization
    - The manuscript is well-structured, with clear sections for introduction, literature review, methods, results, and discussion.

Weaknesses and Recommendations:

1. Research Gaps and Literature Review:
    - While the literature review is comprehensive, it could benefit from including more recent global studies to strengthen the broader context of the research.
    - Some referenced studies (e.g., Xia et al., 2023; Bertrandias et al., 2023) are mentioned superficially without a detailed explanation of how they relate to this study's hypotheses.

2. Theoretical Framework:
    - Although the use of Social Cognitive Theory and the Theory of Planned Behavior is appropriate, the linkage between these theories and the empirical findings could be more explicit.

3. Discussion of Results:
    - The manuscript largely confirms expected relationships (e.g., parental monitoring reduces risks, positive attitudes mediate effects). However, it does not critically explore potential anomalies or unexpected findings, which could add richness to the discussion.
    - The policy recommendations, while useful, are somewhat general. More specific, culturally tailored recommendations for China could enhance the manuscript's practical value.

4. Limitations:
    - The study is cross-sectional, which limits causal interpretations. While this is acknowledged, it should be emphasized more prominently in the discussion.
    - The sample's urban-rural distribution is balanced, but there could be greater acknowledgment of potential socioeconomic disparities beyond parental income.

5. Writing and Presentation:

- Minor grammatical errors and occasional awkward phrasing can be improved for readability and professionalism.
- Figures and tables are not fully integrated into the discussion, leaving readers to infer some connections.

Publication Recommendation:

Conditional Acceptance: The manuscript has significant merit, addressing a timely and relevant topic with strong methodological underpinnings. However, to enhance its impact, the following revisions should be considered before publication:

- Strengthen the literature review with additional context and recent studies.
- Deepen the discussion, focusing on unexpected findings and practical applications tailored to the Chinese context.
- Address the limitations more explicitly and suggest avenues for longitudinal studies.
- Improve the integration of figures and tables with the text and polish the language for clarity and fluency.
