# Supplementary material for: Assessing the relationship between digital technology use and physical health, fitness, and exercise levels among Chinese youth: The moderating effect of parental monitoring
Source: PLoS One. 2025 Jun 4;20(6):e0324705. doi: 10.1371/journal.pone.0324705 (PMC12136296; doi:10.1371/journal.pone.0324705)
Supplement: Attachment — Submitted filename: Response to editor.docx [file pone.0324705.s001.docx]

Dear Editor,

We would like to express our sincere gratitude for your thorough review and valuable comments on our manuscript. We have uploaded the original scanned copy of ethical approval letter in Mandarin, also we have stated the date where our survey conducted and all our accessed data analyzed for this research purpose in the Methods section. All the amended parts have been highlighted. Kindly review these changes. The requested documents have been submitted along with our revised manuscript, thanks a lot.

Regards

Jiajia Cui

Jilin Sports University,

2476 Ziyou Road,

Changchun City,

Jilin Province,

130022, China
