# Supplementary material for: Assessing the relationship between digital technology use and physical health, fitness, and exercise levels among Chinese youth: The moderating effect of parental monitoring
Source: PLoS One. 2025 Jun 4;20(6):e0324705. doi: 10.1371/journal.pone.0324705 (PMC12136296; doi:10.1371/journal.pone.0324705)
Supplement: Attachment — Submitted filename: PONE-D-24-25126.docx [file pone.0324705.s002.docx]

The submitted manuscript (PONE-D-24-25126) entitled “*Assessing the Relationship between Digital Technology Use and Physical Health, Fitness, and Exercise Levels among Chinese Youth: The Moderating Effect of Parental Monitoring*” intends to investigate how digital media use, parental supervision, and attitudes towards physical activity influence young Chinese individuals' physical health and exercise levels. According to my understanding, physical health is the outcome of this work, but who will benefit from the results? The authors did not clarify properly, this is my main concern. The author has made a good effort to investigate the direct effects of social media use, digital technologies, attitudes toward physical activities, educational technology use, and online gaming towards Physical health by collecting data from 827 china residents. My other concerns are given below to improve the current work.

1. The introduction of this work is very poor. The authors should provide a real example for this work to introduce the significance of this work. To create the credibility of the work, the author should propose research questions in the introduction. Authors may reference some articles to provide an interesting intro such as “The COVID-19 Pandemic and Overall Wellbeing: Mediating Role of Virtual Reality Fitness for Physical-Psychological Health and Physical Activity”, ” Demographic Characteristics and Digital Platforms for Physical Activity Among the Chinese Residents During the COVID-19 Pandemic: A Mediating Analysis”, ” Virtual Reality Fitness (VRF) for Behavior Management During the COVID-19 Pandemic: A Mediation Analysis Approach”, “Fitness Apps, Live Streaming Workout Classes, and Virtual Reality Fitness for Physical Activity During the COVID-19 Lockdown: An Empirical Study”, ”Investigating Effects of Digital Innovations on Sustainable Operations of Logistics: An Empirical Study”, ” Physical Activity is a Medicine for Non-Communicable Diseases: A Survey Study Regarding the Perception of Physical Activity Impact on Health Wellbeing”, and ” Sustainable Development Goals, Sports and Physical Activity: The Localization of Health-Related Sustainable Development Goals Through Sports in China: A Narrative Review”.
2. In the literature review section, the theoretical development is poor for the proposed model which should be the 2.1 heading of the literature review section. On what logic the author has proposed the hypothesis? Provide explanations. In addition, The proposed hypotheses are not similar to the conceptual model, authors should modify the figure or all hypotheses. (The names of constructs should be the same in the whole manuscript, especially in keywords).
3. How did you calculate the actual sample size, please provide a reference related to sample size.
4. How about controls for this study? The authors should justify why they did not mention about controls while analyzing the proposed relations.
5. The discriminant and convergent validity of the data is missing in factor analysis. The examination of the reliability tests is poor too and needs improvements further such as calculate the AVEs, SCRs, Alfas, and cross-loadings for each item.
6. In table 5. What is an indirect effect (mediation) please clarify about it.
7. In Table 4, please provide the hypothesis number for each estimate. Similarly, clarify the explanations too.
8. Table 6 has similar issue, there are four moderating effect results, but the study has only H1, and H2, and H3. Even in Figure 1, there are 8 effects among 7 constructs. Figure 1 have not any mediating and moderating effects. Please show the mediating and moderating effects in figure 1 too for clarification.
9. Provide three real examples for research implications in discussion section.

In The conclusion section, the author should introduce a novel construct for further investigations with real examples.
